# An M2e-based multiple antigenic peptide vaccine protects mice from lethal challenge with divergent H5N1 influenza viruses

**DOI:** 10.1186/1743-422X-7-9

**Published:** 2010-01-18

**Authors:** Guangyu Zhao, Yongping Lin, Lanying Du, Jie Guan, Shihui Sun, Hongyan Sui, Zhihua Kou, Chris CS Chan, Yan Guo, Shibo Jiang, Bo-Jian Zheng, Yusen Zhou

**Affiliations:** 1 State Key Laboratory of Pathogen and Biosecurity, Beijing Institute of Microbiology and Epidemiology, Beijing 100071, China; 2 Department of Microbiology, The University of Hong Kong, Hong Kong, China; 3 Lindsley F Kimball Research Institute, New York Blood Center, New York, NY 10065, USA

## Abstract

**Background:**

A growing concern has raised regarding the pandemic potential of the highly pathogenic avian influenza (HPAI) H5N1 viruses. Consequently, there is an urgent need to develop an effective and safe vaccine against the divergent H5N1 influenza viruses. In the present study, we designed a tetra-branched multiple antigenic peptide (MAP)-based vaccine, designated M2e-MAP, which contains the sequence overlapping the highly conserved extracellular domain of matrix protein 2 (M2e) of a HPAI H5N1 virus, and investigated its immune responses and cross-protection against different clades of H5N1 viruses.

**Results:**

Our results showed that M2e-MAP vaccine induced strong M2e-specific IgG antibody responses following 3-dose immunization of mice with M2e-MAP in the presence of Freunds' or aluminium (alum) adjuvant. M2e-MAP vaccination limited viral replication and attenuated histopathological damage in the challenged mouse lungs. The M2e-MAP-based vaccine protected immunized mice against both clade1: VN/1194 and clade2.3.4: SZ/406H H5N1 virus challenge, being able to counteract weight lost and elevate survival rate following lethal challenge of H5N1 viruses.

**Conclusions:**

These results suggest that M2e-MAP presenting M2e of H5N1 virus has a great potential to be developed into an effective subunit vaccine for the prevention of infection by a broad spectrum of HPAI H5N1 viruses.

## Background

The re-emergence of H5N1 highly pathogenic avian influenza (HPAI) in 2003 has caused 262 fatal cases among a total of 442 infected individuals [1]. Therefore, there is an urgent need to develop safe and effective antiviral strategies for the prevention of any future pandemic of H5N1 HPAI [2,3], among which vaccination is still the most effective means to prevent influenza A virus infection. Due to current vaccine technologies facing annual problems with vaccine-strain matching, some conserved antigens of influenza A virus become promising target for the development of influenza vaccines with broad cross-protection.

In comparison with other surface proteins of H5N1 viruses, matrix protein 2 (M2) is the most conserved. The native M2 protein exists as a homotetramer formed by two disulfide linked dimers, with each monomer consisting of 97 amino acids [4,5]. The 24-amino-acid extracellular domain of M2 protein (M2e) is remarkably conserved across influenza A subtypes [6]. Passively transferred anti-M2 monoclonal antibodies (mAbs) accelerated lung viral clearance [7], and mAbs recognizing the N-terminus highly conserved epitope in M2e protected mice from lethal influenza A virus challenge [8], implying that M2, in particular M2e, may serve as an attractive vaccine target. Currently, the rapid evolution of H5N1 virus and the co-circulation of multiple antigenic variants in multiple regions determine that development of H5N1 vaccine with cross-protection against divergent H5N1 viruses would be inevitable. Although a number of M2e-based vaccines have been reported to provide broad-spectrum protection against ordinary human influenza virus infection [9-13], the cross-protective effect to divergent H5N1 viruses was undocumented.

In the present study, we designed and synthesized a tetra-branched multiple antigenic peptide (MAP) derived from the M2e sequence of H5N1 virus VN/1194 strain, denoted as M2e-MAP, with an aim to develop a M2e-based vaccine for induction of M2e-specific immune responses and cross-protection of the vaccinated animals against lethal challenge of divergent H5N1 virus strains.

## Results

### M2e-MAP immunization induced potent M2e-specific antibody responses

**Figure 1:**
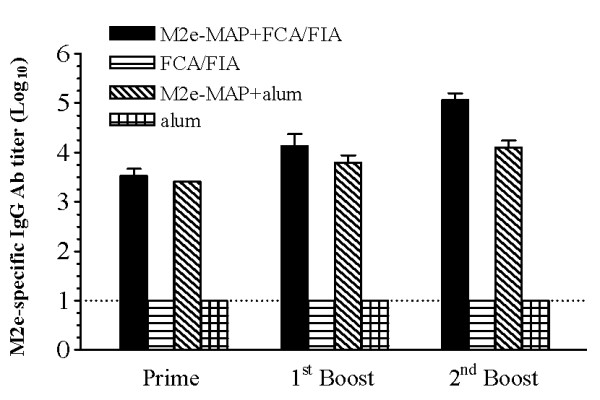
**M2e-specific antibody responses induced by M2e-MAP vaccine**. Mice were vaccinated with M2e-MAP plus FCA/FIA (s.c.) or alum (i.m.) adjuvant for a total of 3 times. Mice receiving FCA/FIA or alum alone were served as adjuvant controls. Mouse sera were collected pre-immunization and 10 days post-each immunization for detection of M2e-specific antibodies by ELISA. The end-point titer of each sample was determined as the highest dilution that yielded an OD_450 nm _value greater than twice that of similarly diluted serum sample collected pre-vaccination. The data are expressed as geometric mean titer (GMT) ± standard deviation (SD) of 10 mice per group. The lower limit detection (1:10) is indicated by a dotted line. Experiments were repeated three times.

To evaluate humoral immune responses potentially induced by M2e-MAP, mice were vaccinated with 10 μg of M2e-MAP plus Freund's or aluminium (alum) adjuvant as described in Methods, and M2e-specific IgG antibodies were detected in mouse serum samples by ELISA. As shown in Figure 1, M2e-MAP induced strong M2e-specific IgG antibody responses, with the highest titer reaching 1:10^5 ^and 1:10^4 ^for M2e-MAP+FCA/FIA and M2e-MAP+alum, respectively, at 10 days post last boost vaccination. In contrast, only background level of antibody responses was detected in the mice receiving adjuvant alone.

### M2e-MAP vaccination limited viral replication and attenuated virus-induced lung pathology

**Figure 2:**
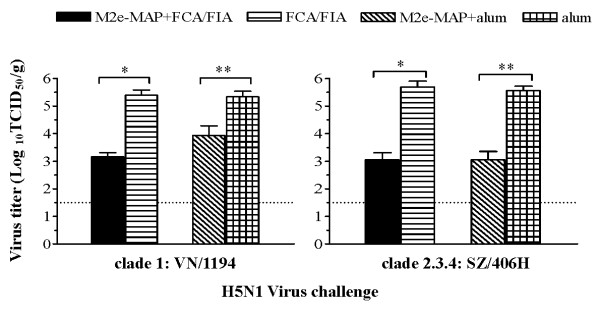
**Virus titers in challenged mouse lungs**. Two weeks post-last boost, mice were challenged with 10LD_50 _of H5N1 virus, clade1: VN/1194 (A.) or clade2.3.4: SZ/406H (B.), and lungs were collected for detection of virus titer five days later. The data are expressed as Log_10_TCID_50_/g of lung tissues. The lower limit of detection is 1.5 Log_10_TCID_50_/g of tissues as indicated by a dotted line. The data are presented as GMT ± SD of 5 mice per group. * indicates *P *< 0.0001 compared to the FCA/FIA control group; ** means *P *< 0.0001 compared to the alum control group. Experiments were repeated three times.

Two phylogenetically distinguished H5N1 virus isolates, clade1: VN/1194 and clade2.3.4: SZ/406H were selected to evaluate the protective immunity afforded by M2e-MAP vaccine *in vivo*. Two weeks after the last boost, mice were challenged with 10 LD_50 _of H5N1 virus VN/1194 or SZ/406H. Five days post-challenge, lungs were removed from infected mice, and infectious virus titers in the lung tissues were measured to determine the protective effects of M2e-MAP vaccine on viral clearance. Compared with those from the adjuvant control (FCA/FIA or alum), the virus titers in the lungs of M2e-MAP vaccinated mice were significantly lower after H5N1 virus challenge (*P *< 0.0001) (Figure 2), suggesting that M2e-MAP vaccine can induce protective immunity against viral replication in vaccinated mice.

**Figure 3:**
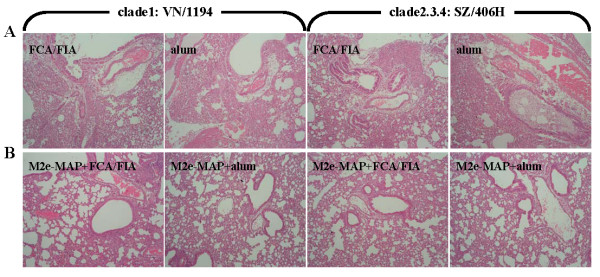
**Histopathological changes in the lungs of virus challenged mice**. Mouse lungs were collected for histopathological analysis 5 days after virus challenge. The figure indicates the representative images of histopathological damage from H&E-stained lungs of mice vaccinated with M2e-MAP plus adjuvant or adjuvant only (magnification, 100×).

Further examination of the lung tissues of virus-challenged mice revealed dramatic histopathological damage in the pulmonary airways and parenchymal tissues in the adjuvant group, including severe damage of bronchial epithelium with necrosis and desquamation, pulmonary vascular dilatation and congestion, infiltration of prominent number of lymphocytes accompanied by exudates and severe edema, especially around vessels, as well as broadening interstitial spaces or fused alveoli walls with focal hemorrhage (Figure 3A). In contrast, lungs of M2e-MAP-vaccinated mice exhibited less histopathological changes, accompanied by only mild pulmonary interstitial pneumonia and moderate lymphocytic infiltration (Figure 3B). The above data implied that M2e-MAP vaccination may protect the mice against lethal challenge of divergent H5N1 viruses through a combination of limiting viral replication in the lungs and attenuating virus-induced lung pathology.

### M2e-MAP vaccination elicited cross-protection against lethal challenge of divergent H5N1 viruses

After receiving the lethal dose (10 LD_50_) of two H5N1 virus strains, the M2e-MAP vaccinated mice were further evaluated in terms of cross-protective ability by daily observation of the clinical symptoms, including weight loss and survival rate for two weeks, and then histopathological examination following removal of lung tissues.

**Figure 4:**
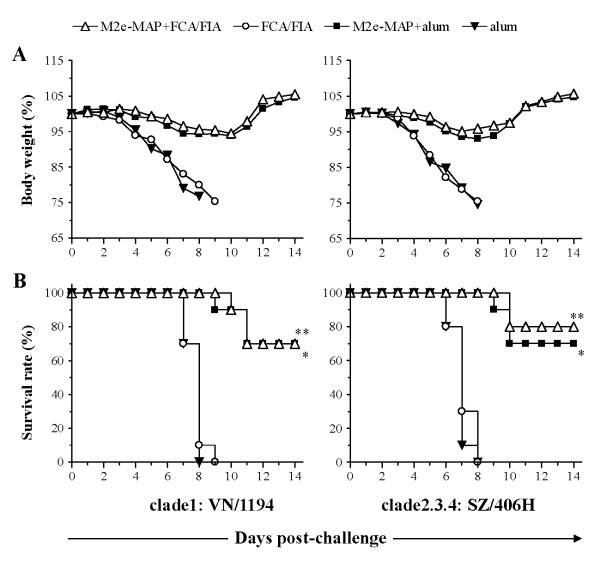
**Weight loss and survival in lethal H5N1 virus challenged mice**. Mice were challenged with 10LD_50 _of H5N1 virus, clade1: VN/1194 or clade2.3.4: SZ/406H, and monitored daily for 2 weeks post-challenge. A. Percentage change (%) of mouse body weight. Each point represents mean body weight of 10 mice per group. B. Survival rate. The significant differences (*P *< 0.0001) of M2e-MAP+FCA/FIA versus FCA/FIA and M2e-MAP+alum versus alum are indicated as * and **, respectively.

From day 4 after VN/1194 and day 3 after SZ/406H virus infection, the adjuvant control mice (FCA/FIA or alum) developed obvious clinical signs, including ruffled fur, hunched posture, rapid breathing, inactivity and paralysis of posterior limb. These clinical signs were either not observed or were delayed for 2-3 days in the M2e-MAP vaccination group. Most M2e-MAP vaccinated mice with clinical signs only exhibited slight or partial piloerection for 2-3 days and then recovered. Moreover, compared to the adjuvant control, M2e-MAP vaccinated mice that did not survive the challenges were observed to have delayed the onset of illness and longer survival days. The observed protection against clinical signs correlated with the changes in body weight. In the adjuvant group, body weight dramatically decreased and even reached a near 25% severe weight loss after H5N1 virus infection. In contrast, the average body weight in the M2e-MAP vaccination group only slightly decreased (less than 10%) during 6-10 days after challenge and then steadily increased (Figure 4A).

All control mice receiving FCA/FIA or alum adjuvant alone died from virus challenge, while 70% to 80% of the mice vaccinated with M2e-MAP survived the lethal H5N1 virus challenge (Figure 4B), with significantly increased survival rate (*P *< 0.0001). At 14 days post-challenge, lung tissues from surviving mice were removed to evaluate viral replication and histopathological damage. No H5N1 virus was detected in the lungs of surviving mice, and the lung tissues of these mice presented almost normal structures (data not shown). The above data confirmed that M2e-MAP vaccine can afford cross-protection against lethal challenge of divergent H5N1 viruses.

## Discussion

A cross-protective vaccine for antigenic variants of H5N1 virus is a very important component in prophylactic strategy against a possible human pandemic. Although the highly conservative M2e of influenza A virus is one of the most promising target for development of universal influenza vaccines, some strategies would be required to improve immunogenicity of vaccines based on M2e containing only 24 amino acid.

The high molar ratio and dense packing of multiple copies of a target antigen in MAP system have been shown to stimulate better immune responses than single-chain peptides [14-16]. Mozdzanowska et al. [17] reported that MAP system presenting human influenza M2e sequence was an effective immunogen, with anti-M2e IgG antibodies from mice immunized with M2e-MAP binding specifically to M2-expressing cells. However, it is unclear whether M2e-MAP could be used as a vaccine candidate to provide effective protection against HPAI H5N1 virus. Different from human influenza A viruses of H1, H2 or H3, H5N1 viruses have circulated only in domestic and wild birds so far [18-21]. The H5N1 viruses leading to human infections still belong to the avian type [22]. M2e vaccine designed on sequences of human influenza virus H1, H2 or H3 subtype may not provide the same protection against H5N1 virus leading to human infections [11]. Moreover, the 10-20 amino acid region of M2e was consistent with host restriction specificities [23].

**Figure 5:**
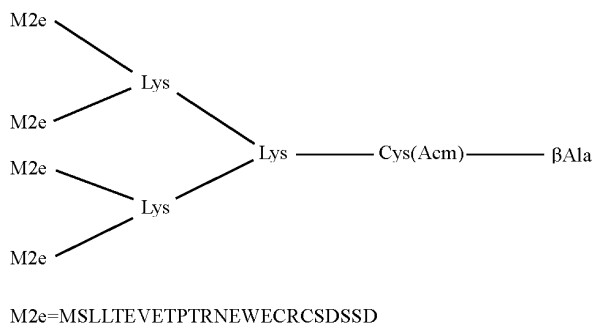
**Construction and sequence of M2e-MAP**. The final form of M2e-MAP was synthesized on [Fmoc-Lys(Fmoc)]2-Lys-Cys(Acm)-βAla-Wang Resin in tetra-branched form, which carries four copies of M2e peptide. The sequence of M2e from highly pathogenic avian influenza H5N1 virus VN/1194 strain is shown at the bottom.

In this study, we designed M2e-MAP in tetra-branched form containing 4 copies of sequence overlapping M2e of VN/1194, a HPAI H5N1 strain (Figure 5). High titers of M2e-specific antibody responses could be induced following immunization of M2e-MAP plus Freund's adjuvant, a commonly used adjuvant in animal experiments or alum, a common adjuvant for human vaccines (Figure 1). Although it has been shown that M2e-specific IgG antibodies do not exhibit an ability to directly neutralize virus *in vitro*, the antiviral effect of M2e-based vaccines was mediated by antibodies to M2e antigen. The mechanism of such antibody mediated antiviral effect could be due to antibody-dependent cell-mediated cytotoxicity (ADCC) and/or complement-mediated cytotoxicity (CDC) [24,25]. Therefore, the induction of M2e-specific antibody responses was necessary for M2e-based vaccines in the prevention of H5N1 virus infection.

Strikingly, M2e-MAP vaccination with Freund's and alum adjuvants conferred cross-protection against a lethal challenge with not only the homologous strain, VN/1194 in clade1, but also a divergent strain, SZ/406H in clade2.3.4. M2e-MAP vaccination in mice delayed the onset of illness, prolonged survival, alleviated body weight loss, limited viral replication and restricted histopathological damage in lung tissues (Figure 2, Figure 3 and Figure 4). The clade categorization of the VN/1194 and SZ/406H strains was based on the phylogenetic analysis of the HA gene. The clade2.3.4 viruses seem to have been prevalent in China since late 2005 and were also responsible for human infection in Laos, Malaysia and Vietnam [26,27], which included A/Hunan/2/2009, one of the most recent human isolates from China [28].

Compared with the monomeric M2e peptide-based vaccines, M2e-MAP vaccine could induce stronger protective immunity due to the immune enhancing effect of the T-helper epitope in the vaccine [14]. In terms of the safety, the synthetic peptide-based M2e-MAP vaccine is superior to the DNA-, adenoviral vector-, and recombinant protein-based M2 vaccines. For example, DNA vaccines have the potential to integrate the viral genomes into human DNA, to develop autoimmunity, and to induce antibiotic resistance [29]. The recombinant protein-based vaccine may have contamination of endotoxin and other unwanted antigens from cell cultures [30]. In spite of a relatively higher cost of production, synthetic peptide-based vaccine can be rapidly designed and synthesized and more convenient for storage and transportation, making M2e-MAP vaccine a better choice for speed development of effective and safe vaccines to combat emerging influenza pandemic.

Based on the evidence of our findings, the approach of developing M2e-based vaccines with broad cross-protection is feasible. Some strategies, such as incorporation of appropriate adjuvants into the present MAP system, modification of the present M2e consensus sequences of H5N1 viruses as vaccine target, and/or selection of other M2e expression forms, might be helpful to achieve full cross-protection against lethal H5N1 virus infection. The effects of such approaches need to be determined in further studies. The data reported here support the concept of developing an M2e-based vaccine candidate that could provide cross-protection against divergent HPAI H5N1 viruses. Such vaccine based on the highly conserved target antigen of H5N1 viruses might be used in combination with current H5N1 influenza vaccines to enhance protection and to prevent a possible future human pandemic of H5N1 influenza.

## Conclusions

In the present study, we synthesized tetra-branched M2e-MAP based on the M2e sequence of H5N1 virus. The M2e-MAP vaccine induced strong M2e-specific IgG antibody responses, being able to protect mice against lethal challenge of both clade1: VN/1194 and clade2.3.4: SZ/406H H5N1 viruses. Based on the evidence of our findings, the approach of developing M2e-based vaccine with broad cross-protection is feasible. The data reported here support the concept of developing an M2e-based vaccine that could provide cross-protection against divergent HPAI H5N1 viruses.

## Methods

### Tetra-branched M2e multiple antigenic peptide (M2e-MAP)

Tetra-branched M2e-MAP carrying four copies of M2e peptide of H5N1 virus strain VN/1194 was synthesized on [Fmoc-Lys(Fmoc)]_2_-Lys-Cys(Acm)-βAla-Wang Resin (Advanced ChemTech, Louisville, Kentuchy, USA) on a 0.02 mM scale using an Applied Biosystems model 433A peptide synthesizer. Cleavage of the peptide from the resin was performed by treatment with trifluoroacetic acid (TFA), DTT, water, and triisopropylsila (TIPS) in the ratio 88:5:5:2 (TFA/DTT/H_2_O/TIPS). Crude peptide was purified by reversed phase high-performance liquid chromatography (RP-HPLC). The purified peptide was characterized by amino acid analysis and matrix-assisted laser desorption ionization mass spectrometry (MALDI-MS). Figure 5 shows the composition and sequence of M2e-MAP.

### Mice

Female BALB/c mice at 4-6 weeks were purchased from the Laboratory Animal Unit and housed in the animal facility of The University of Hong Kong following the approved animal care protocols. Mice were rested for 2 weeks before immunization. The animal study was approved by the Department of Health of Government of Hong Kong Special Administration Region, and University Animal Ethics Committee of The University of Hong Kong.

### Viruses

HPAI H5N1 virus isolates used in this study were clade1: VN/1194 and clade2.3.4: SZ/406H. H5N1 viruses were grown in the allantoic cavities of 10-day-old embryonated chicken eggs. Virus-containing allantoic fluid was harvested and stored in aliquots at -80°C until use. The LD_50 _of each virus stain was determined in mice after serial dilutions of the virus stock. All infectious experiments related to H5N1 viruses were performed in an approved biosafety level 3 (BSL-3) facility at The University of Hong Kong.

### Animal experiment

Mice were subcutaneously (s.c.) prime vaccinated with M2e-MAP (10 μg/mouse) plus Freund's complete adjuvant (FCA, sigma) and boosted twice with the same amount of immunogen plus Freund's incomplete adjuvant (FIA, Sigma) at 3-week intervals. For parallel experiments, mice were also intramuscularly (i.m.) vaccinated with the same amount of M2e-MAP immunogens plus alum adjuvant (Sigma) at the same condition of the above. Mice injected with Freund's or alum adjuvant were used as the respective control. Mouse sera were collected before immunization and 10 days post-each vaccination for detection of antibody responses.

Two weeks post-last vaccination, mice were intraperitoneally (i.p.) anesthetized with ketamine-xylazine (75/5 mg/kg), and intranasally (i.n.) challenged with 10LD_50 _of clade1: VN/1194 or clade2.3.4: SZ/406H H5N1 virus stain. Infected mice were observed and weighed daily for 2 weeks. Lung tissues were collected from euthanized mice 5 days post-challenge for further virological test and histopathological analysis.

### ELISA

The M2-specific IgG antibody in the vaccinated mouse sera was detected by ELISA as previously described [31] with some modifications. Briefly, 96-well microtiter plates were pre-coated with 5 μg/ml of M2e-MAP overnight at 4°C. After blocking with 3% BSA containing 0.05% Tween-20 in PBS, serial diluted mouse sera were added to the plates, followed by adding HRP-conjugated rabbit anti-mouse IgG (1:2,000, Invitrogen, Carlsbad, CA) for 1 h at 37°C. Assay was developed using 3,3',5,5'-tetramethylbenzidine (TMB) (Zymed, Carlsbad, CA), and the reaction was stopped by adding 1N H_2_SO_4_. The absorbance at 450 nm was measured by an ELISA plate reader (Sunrise™ microplate reader, TECAN, NC).

### Virus titers in lung tissues

Lung tissues from euthanized mice were aseptically removed and homogenized in minimal essential medium (MEM) plus antibiotics to achieve 10% (w/v) suspensions of lungs. Ten-fold serial dilutions of samples were added in quadruplicate to the monolayer of Madin-Darby canine kidney (MDCK) cells seeded at 96-well cell culture plates 12 h before infection, and allowed to absorb for 2 h at 37°C. Fresh medium was then added to the cells and continued to incubate for 72 h. Virus cytopathic effect (CPE) was observed daily and the viral titer was determined by the hemagglutinin (HA) test indicated as follows. Fifty microlitre of 0.5% turkey red blood cells (Lampire Biological Laboratories, Pipersville, PA) was added to 50 μl of cell culture supernatant and incubated at room temperature for 30 min. Wells containing HA were scored as positive. The virus titer was calculated by the Reed and Muench method and expressed as Log_10_TCID_50_/g of lung tissues.

### Histopathological analysis

The lung tissues of challenged mice were immediately fixed in 10% neutral buffered formalin and embedded in paraffin wax. Sections were made at 4-6 μm thickness and mounted on slides. Histopathological changes were examined by H&E staining and observed under light microscopy as previously described [32].

### Statistical analysis

The significance between survival curves was analyzed by Kaplan-Meier survival analysis with log-rank test. Other data were analyzed using the 2-tailed Student's t test. *P *< 0.0001 was considered significant. All analyses were performed in Graphpad Prism software.

## Abbreviations

HPAI: highly pathogenic avian influenza; M2e-MAP: M2e-based multiple antigenic peptide; FCA: Freunds' complete adjuvant; FIA: Freunds' incomplete adjuvant; VN/1194: A/Vietnam/1194/2004(H5N1); SZ/406H: A/Shenzhen/406H/2006(H5N1); LD_50_: 50% lethal dose; Log_10_TCID_50_/g: Log_10 _50% tissue-culture infectious dose per gram.

## Competing interests

The authors declare that they have no competing interests.

## Authors' contributions

GZ, BZ and YZ designed research. GZ, YL, JG, SS, HS and CC performed research. GZ, SS, ZK and YG analyzed data. GZ, LD, SJ, BZ and YZ wrote and modified the paper. All authors read and approved the final manuscript.
